# MicroRNA-27a/b-3p and PPARG regulate SCAMP3 through a feed-forward loop during adipogenesis

**DOI:** 10.1038/s41598-019-50210-3

**Published:** 2019-09-25

**Authors:** Agné Kulyté, Kelvin Ho Man Kwok, Michiel de Hoon, Piero Carninci, Yoshihide Hayashizaki, Peter Arner, Erik Arner

**Affiliations:** 1 Lipid laboratory, Department of Medicine H7, Karolinska Institutet, Huddinge, Sweden; 2 Department of Biosciences and Nutrition, Karolinska Institutet, Huddinge, Sweden; 3 RIKEN Center for Integrative Medical Sciences (IMS), Yokohama, Kanagawa 230-0045 Japan; 4 RIKEN Preventive Medicine and Diagnosis Innovation Program, Yokohama, Kanagawa 230-0045 Japan

**Keywords:** Endocrinology, Medical research

## Abstract

MicroRNAs (miRNA) modulate gene expression through feed-back and forward loops. Previous studies identified miRNAs that regulate transcription factors, including Peroxisome Proliferator Activated Receptor Gamma (PPARG), in adipocytes, but whether they influence adipogenesis *via* such regulatory loops remain elusive. Here we predicted and validated a novel feed-forward loop regulating adipogenesis and involved miR-27a/b-3p, PPARG and Secretory Carrier Membrane Protein 3 (SCAMP3). In this loop, expression of both PPARG and SCAMP3 was independently suppressed by miR-27a/b-3p overexpression. Knockdown of PPARG downregulated SCAMP3 expression at the late phase of adipogenesis, whereas reduction of SCAMP3 mRNA levels increased PPARG expression at early phase in differentiation. The latter was accompanied with upregulation of adipocyte-enriched genes, including ADIPOQ and FABP4, suggesting an anti-adipogenic role for SCAMP3. PPARG and SCAMP3 exhibited opposite behaviors regarding correlations with clinical phenotypes, including body mass index, body fat mass, adipocyte size, lipolytic and lipogenic capacity, and secretion of pro-inflammatory cytokines. While adipose PPARG expression was associated with more favorable metabolic phenotypes, SCAMP3 expression was linked to increased fat mass and insulin resistance. Together, we identified a feed-forward loop through which miR-27a/b-3p, PPARG and SCAMP3 cooperatively fine tune the regulation of adipogenesis, which potentially may impact whole body metabolism.

## Introduction

Adipocytes are the specific cellular components of white adipose tissue (WAT), an organ important for energy balance as well as endocrine and paracrine signaling. New fat cells are generated through adipogenesis which is essential for growth of fat mass^1^. It is a two-step process in which an undifferentiated mesenchymal cell differentiates into a preadipocyte, which then undergoes a secondary differentiation step to become a lipid-filled adipocyte^2^. Adipogenesis is tightly controlled by many signaling molecules and transcription factors. Among the latter, peroxisome proliferator activated receptor gamma (PPARG) is recognized as the master regulator of adipocyte differentiation as reviewed^3^. PPARG is both sufficient and necessary for the differentiation of white adipocytes^4^.

Other adipogenic regulators involve microRNAs (miRNAs). They are a class of short non-coding RNAs acting at the posttranscriptional level in most cell types and also playing an important role in the regulation of adipocyte differentiation and mature adipocyte function. A number of adipocyte-selective miRNAs have been described to interact with transcription factors regulating adipogenesis including PPARG as discussed in detail^5^. A particular miRNA typically targets multiple mRNAs creating room for a complex regulation (stimulation or inhibition). In many cell types miRNAs form feed-back or feed-forward loops to fine tune regulation of gene expression^6^. The role of such loops in adipogenesis is less well understood. We recently investigated the transcriptional dynamics during human adipogenesis and found that different types of transcripts (coding genes, enhancers, long noncoding RNAs) showed common dynamics during fat cell differentiation^7^.

 Feed-forward loops (FFLs) are a common feature of transcriptional networks^8^, and FFLs involving miRNAs are recurring motifs in mammalian cells^9^ that may serve as a way of controlling the levels of transcription factors (TFs) and their targets^10^. FFLs may play important roles in differentiation^11,12^ but it is not clear to what extent this network motif is present in adipogenesis. With this in mind, we set up to identify and experimentally verify miRNA–TF–target gene feed-forward loops involving PPARG during human adipocyte differentiation *in vitro*.

## Results

### Construction of feed-forward loops during adipogenesis

**Figure 1:**
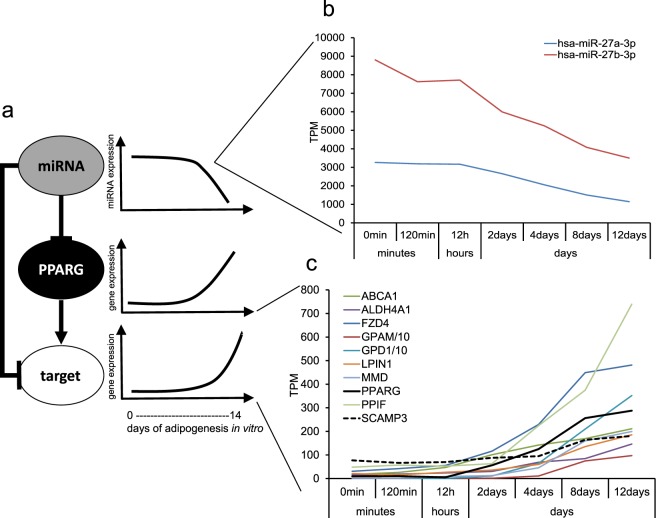
A concept figure for the study. (**a**) A pipeline implemented to elucidate miRNA–PPARG-target regulatory networks during human adipogenesis. T-bars indicate inhibition; arrows indicate stimulation. (**b,c**) Expression of miR27a/b-3p and ten predicted targets of miR-27b-3p during adipogenesis. TPM values of GPAM and GPD1 were divided by 10-fold to facilitate visualization in the same graph.

In order to construct a list of potential FFLs consisting of PPARG, a miRNA and a target gene, we searched FANTOM5 adipogenesis CAGE^7^ and miRNA^13^ time course data for highly expressed miRNAs (>=10000 tags per million (TPM)) and genes (promoter expression >= 150 TPM) predicted to be targets of the miRNA according to the TargetScan method^14^. The fat cells and their precursors in FANTOM5 were generated and differentiated in our laboratory using the protocol described in Material and Methods. Considering that PPARG was chosen as the transcription factor central to the loop in this screening for FFL candidates, and that PPARG is a positive regulator of gene expression and is upregulated during adipogenesis, we required that the miRNA and the target gene were at least two-fold downregulated (miRNA) or upregulated (target gene) during adipocyte differentiation, and that their expression profile correlation (r-value) across the time course was −0.95 or lower. Finally, we required that PPARG was a predicted target of the miRNA, and that the miRNA target gene also was a predicted target of PPARG based on previously published ChIP-seq data^15^ (Fig. 1a). Using these requirements, we obtained a list of ten candidate FFLs where a miRNA was predicted to regulate PPARG as well as a target gene of PPARG, all with miR-27b-3p as the candidate miRNA regulator (Fig. 1b,c and Table 1). Previous studies have shown that miR-27a/b (Table 2) directly binds to the 3′UTR of PPARG, which decreases its expression and consequently inhibits adipogenesis. Consistent with this role, miR-27a/b-3p expression is down-regulated during adipogenesis^16^. The same was observed by us, as miR-27a/b-3p were downregulated about 60 to 65% during adipogenesis in the FANTOM5 data^13^ (Fig. 1b).

**Table 1:** Differentially expressed miRNAs and their predicted targets during adipogenesis.

miRNA	Genes	Gene name
hsa-miR-27b-3p	ABCA1	ATP Binding Cassette Subfamily A Member 1
hsa-miR-27b-3p	ALDH4A1	Aldehyde Dehydrogenase 4 Family Member A1
hsa-miR-27b-3p	FZD4	Frizzled Class Receptor 4
hsa-miR-27b-3p	GPAM	Glycerol-3-Phosphate Acyltransferase, Mitochondrial
hsa-miR-27b-3p	GPD1	Glycerol-3-Phosphate Dehydrogenase 1
hsa-miR-27b-3p	LPIN1	Lipin 1
hsa-miR-27b-3p	MMD	Monocyte To Macrophage Differentiation Associated
hsa-miR-27b-3p	PPARG	Peroxisome Proliferator Activated Receptor Gamma
hsa-miR-27b-3p	PPIF	Peptidylprolyl Isomerase F
hsa-miR-27b-3p	SCAMP3	Secretory Carrier Membrane Protein 3

**Table 2:** Specifications of miR-27a/b-3p.

Name	Sequence	MirBase ID
hsa-miR-27a-3p	uucacaguggcuaaguuccgc	MIMAT0000084
hsa-miR-27b-3p	uucacaguggcuaaguucugc	MIMAT0000419

### Impact of miR-27a/b-3p on SCAMP3, ABCA1 and PPARG expression

Out of the ten candidate loops, we next picked two for validation in the context of interacting with PPARG. One was a target gene with established function in adipose function, namely ATP Binding Cassette Subfamily A Member 1 (ABCA1). Its expression increases during adipogenesis^17^, and is involved in regulation of cholesterol content in plasma membrane and lipogenesis in adipocytes^18^. In addition to ABCA1 we also picked SCAMP3, a gene with previously unreported role in adipogenesis or adipocyte function. SCAMP3 is a secretory carrier membrane protein and functions as a carrier to the cell surface in post-Golgi recycling pathways^19,20^.

**Figure 2:**
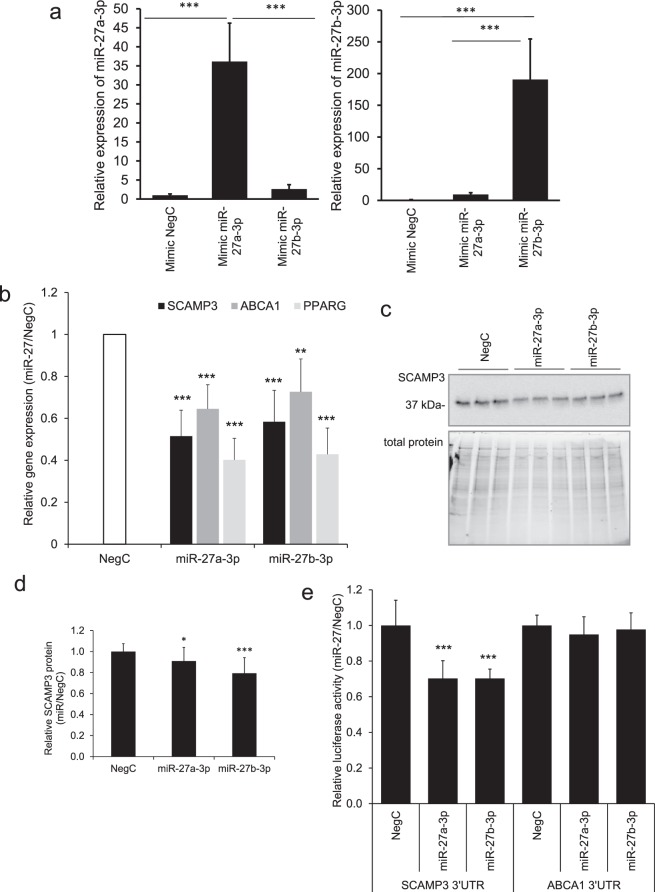
Functional validation of miR-27a/b-3p effects on PPARG, SCAMP3 and ABCA1 in human adipocytes. (**a**) miR-27a-3p and miR-27b-3p were overexpressed in *in vitro* differentiated in hASCs and their expression was assessed by RT-qPCR. Results are based on three biological/independent experiments. Expression of genes was normalized to the reference gene *SNORD68*. (**b**) miR-27a-3p and miR-27b-3p were overexpressed in *in vitro* differentiated in hASCs and expression of PPARG, SCAMP3 and ABCA1 was assessed by RT-qPCR. Results are based on three biological/independent experiments. Expression of genes was normalized to the reference gene *LRP10*. (**c,d**) miR-27a-3p and miR-27b-3p were overexpressed in *in vitro* differentiated in hASCs, cells were lyzed to collect the total protein and thereafter proteins were analyzed by Western blot. Results are based on four biological/independent experiments. Expression of SCAMP3 was normalized to the total protein amount. (**e**) Mimics of miR-27a-3p and miR-27b-3p were transfected together with 3′UTR reporter constructs for SCAMP3, ABCA1 or empty reporter vector in 3T3-L1 cells and changes of luciferase activity was measured in cell lysates. Results are based on two or three biological/independent experiments. Results were analyzed using t-test and presented in fold change ± SD relative to negative control (Neg C). ***P < 0.005, *P < 0.05.

In order to confirm if miR-27a/b-3p affects SCAMP3, ABCA1 and PPARG expression, we overexpressed each miRNA in *in vitro* differentiated human adipose-derived stem cells (hASCs) late during differentiation (day 8) and performed quantification of the miRNA expression and mRNA analysis at the terminal stage of differentiation at day 12 (Fig. 2a,b). Expression of SCAMP3, ABCA1 and PPARG mRNA levels were reduced significantly by, respectively, 50%, 30%, and 60% by each miRNA (Fig. 2b). Effects of the miR-27a/b-3p on SCAMP3 expression were also confirmed at the protein level (Fig. 2c,d).

To verify whether the miR-27a/b-3p bound directly to SCAMP3 and ABCA1 mRNA, we performed a 3′-UTR binding assay where mimics of miR-27a/b-3p were co-transfected together with the 3′-UTR of SCAMP3 or ABCA1 fused to a luciferase reporter. Overexpression of miR-27a/b-3p led to a significant downregulation of luciferase activity for the SCAMP3 reporter, but not for ABCA1 indicating that ABCA1 may not be a direct target of miR-27a/b-3p (Fig. 2e).

Based on public ChIP-Seq data, PPARG regulates SCAMP3 [ChIP-atlas at BioRxiv, 10.1101/262899]. Although we had experimental evidence of direct binding of miR-27a/b-3p to the 3′UTR of SCAMP3, we wanted to ascertain that miR-27a/b-3p indeed regulates SCAMP3 independent of PPARG. To assess this, we overexpressed miR-27a/b-3p in proliferating hASCs (where expression of PPARG is negligible) and evaluated expression of SCAMP3. Indeed, expression of SCAMP3 was significantly downregulated by 30% by each miRNA mimic (Supplemental Fig. 1). Together, this data strongly suggest that miR-27a/b-3p directly regulates SCAMP3, as well as through a FFL with PPARG. In order to elucidate the effects of this regulatory network on adipogenesis and adipocyte function, we focused the rest of the study on SCAMP3.

### Role of SCAMP3 in adipocyte function

**Figure 3:**
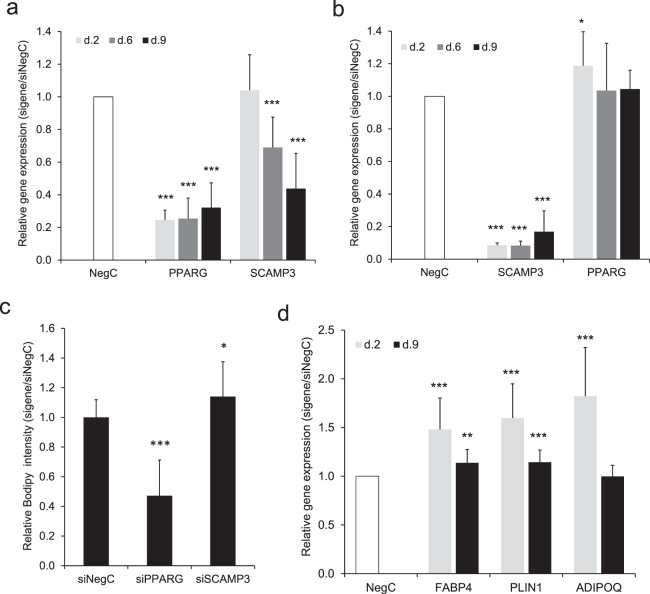
Impact of SCAMP3 on human adipogenesis. (**a**) Expression of PPARG was knocked down using siRNA in hASCs 24 h before induction of differentiation and until days 2, 6 and 9 of differentiation, upon which the expression of PPARG and SCAMP3 was monitored. (**b**) Expression of SCAMP3 was knocked down using siRNA in hASCs 24 h before induction of differentiation until days 2, 6 and 9 of differentiation, upon which the expression of SCAMP3 and PPARG was monitored. (**c**) Expression of SCAMP3 and PPARG was knocked down using siRNA in hASCs 24 h before induction of differentiation until day 9 of differentiation when accumulation of neutral lipids and number of cells was evaluated. (**d**) Expression of SCAMP3 was knocked down using siRNA in hASCs 24 h before induction of differentiation until days 2, 6 and 9 of differentiation, upon which the expression of FABP4, PLIN1 and ADIPOQ was monitored. Results in A-D are based on four biological/independent experiments. Expression of genes was normalized to the reference gene 18 s. Results were analyzed using t-test and presented in fold change ± SD relative to negative control of a corresponding time point (Neg C). ***P < 0.005, **P < 0.01, *P < 0.05.

Since the predicted regulatory loop contained a PPARG-SCAMP3 axis, we investigated if PPARG regulates the expression of SCAMP3 in adipocytes. For this purpose, we transfected hASCs with siRNA targeting PPARG 24 h before induction of differentiation and monitored expression of SCAMP3 during adipogenesis at day 2, 6, and 9. While no effect on SCAMP3 expression was observed at day 2, the mRNA levels of SCAMP3 at day 6 and 9 were significantly reduced, respectively, by 30% and 50% (Fig. 3a). Knockdown of PPARG expression was efficient but, as expected, gradually decreased slightly from 75% to about 70% towards the end of differentiation.

To examine whether SCAMP3 has an impact on PPARG expression during adipogenesis, hASCs were transfected with siRNA targeting SCAMP3 using the same experimental setup and expression of PPARG and SCAMP3 during differentiation was monitored. Knocking down SCAMP3 increased expression of PPARG by 20% at day 2. However, the effect did not last until day 6 and day 9. Efficiency of SCAMP3 knockdown was about 90 to 80% (day 2 to day 9) (Fig. 3b).

To further investigate the role of SCAMP3 in adipocyte development, we performed knockdown of SCAMP3 followed by lipid staining measurement. Knockdown of SCAMP3 before induction of differentiation increased accumulation of neutral lipids by 15% at day 9 (Fig. 3c and Supplemental Fig. 2). Knockdown of PPARG was performed in parallel as a positive control for the assay (Fig. 3c and Supplemental Fig. 2). Indeed, knockdown of PPARG reduced adipocyte differentiation and consequently lipid accumulation by 50%.

To assess whether SCAMP3 has an impact on the adipocyte-specific genes, we applied the similar early knockdown of SCAMP3 followed by the expression measurements of Perilipin 1 (PLIN1), Fatty Acid Binding Protein 4 (FABP4) and Adiponectin (ADIPOQ). Expression of FABP4, PLIN1 and ADIPOQ was increased at day 2 by 50%, 60% and 80%, respectively. Expression of FABP4 and PLIN1 but not ADIPOQ was still increased by 15% at day 9 (Fig. 3d). Together, these results suggest that SCAMP3 has an inhibitory role at the early stage of adipogenesis.

### Association between expression of PPARG, SCAMP3 and adipose tissue parameters

In order to examine the impact of PPARG and SCAMP3 on clinical adipose phenotypes we made a multiple regression analysis of gene expression versus adipose phenotypes using previously generated data from a cohort of 56 women (described in Material and Methods). PPARG and SCAMP3 correlated in an opposite fashion with the adipose phenotypes. High PPARG expression was related to a positive phenotype (low BMI and body fat, small fat cells, high lipogenesis, low secretion of inflammatory proteins and low lipolytic activity). The opposite was observed for SCAMP3, e.g. expression of SCAMP3 correlated with more body fat, insulin resistance *in vivo* and *in vitro*, large fat cells and enhanced lipolysis (Table 3). In summary, the data suggest that adipose PPARG and SCAMP3 have opposite roles for clinical phenotypes and fat cell function when investigated together.

**Table 3:** Correlation between adipose gene expression and clinical or adipose tissue parameters.

Phenotype	PPARG		SCAMP3	
	partial r-value	p-value	partial r-value	p-value
Body mass index, kg/m	−0.82	<0.0001	0.76	<0.00001
Body fat, % of total body weight	−0.72	<0.0001	0.73	<0.0001
Waist circumference, cm	−0.78	<0.0001	0.78	<0.0001
Waist-to-hip, ratio cm/cm	−0.58	0.0003	0.62	0.0001
Homeostasis model assessment of *in vivo* insulin sensitivity, arbitrary units*	−0.56	0.0003	0.62	0.0001
Fat cell volume, picolitres	−0.83	<0.0001	0.73	<0.0001
Insulin stimulated lipogenesis in fat cells, nmoles of glucose/2 hours/10^7^ fat cells*	0.43	0.017	−0.56	0.002
Adipose tissue secretion of interleukin-6, ng/2 hours/10^7^ fat cells*	−0.62	0.001	0.58	0.002
Adipose tissue secretion of tumor necrosis factor alpha, ng/2 hours/10^7^ fat cells*	−0.57	0.002	0.75	<0.0001
Adipose tissue lipolytic activity, µmoles of glycerol/2 hours/10^7^ fat cells*^#^	−0.66	0.007	0.42	0.025

Multiple regression was used. A negative r-value indicates inverse relationship between regressor and phenotype.

*Values were (10) log transformed prior to use. ^#^This is release of glycerol (an end products of triglyceride hydrolysis) into the medium of adipose tissue incubated pieces.

## Discussion

This study proposes that adipogenesis regulation involves miRNA acting through FFLs. We identified such a loop involving miR-27a/b-3p, PPARγ and SCAMP3. There are several ways that miRNAs, TFs and target genes can interact in FFLs. The type we studied here, where the miRNA is a negative regulator upstream of both the TF and its (positively regulated) target, has been termed “coherent type 2”^8^. The proposed function of this type of FFL is to ensure that the miRNA and its downstream target are not expressed simultaneously in the same tissue, their expression patterns thus defining spatiotemporal boundaries between developmental stages^12^. Another proposed function of this type of FFL is to fine tune the ratio between TF and target abundance^21^. This interaction may be particularly relevant here, considering the opposite effects of PPARG and SCAMP3 on adipogenesis and adipocyte related function we observed (further discussed below).

miR-27 has been previously described as anti-adipogenic acting on the PPARG and CCAAT Enhancer Binding Protein Alpha (CEBPA) expression in mice and human fat cell models^22,23^. Multiple targets such as prohibitin, adipogenic transcription factor cAMP response element-binding protein (CREB), lysyl oxidase and thus multiple pathways of adipogenesis have been described to be inhibited by miR-27^24–26^. Treatment with tumor necrosis factor-α (TNFA), one of the key adipokines in obesity and insulin resistance, increases miR-27^25^. Consequently, expression of miR-27 can be linked to inability to increase fat cell number and hence causes a hypertrophic expansion of adipose tissue (increased fat cell size instead of number)^22^.

This study also identified SCAMP3 as a novel direct target of the anti-adipogenenic miR-27a/b-3p. Secretory carrier membrane proteins (SCAMPs) are ubiquitously expressed proteins of post-Golgi vesicles. SCAMP3 has been reported to regulate epidermal growth factor receptor degradation and recycling through the actions of multivesicular bodies^19,20^, but its function in adipocytes or in metabolic context is not known. Based on previously published ChIP-seq data, SCAMP3 is a target of PPARG and we could confirm that knockdown of PPARG downregulated expression of SCAMP3. Importantly, we could demonstrate that miR-27a/b-3p affected SCAMP3 independently of PPARG.

Having established the regulatory connection between PPARG and SCAMP3, we hypothesized that SCAMP3 would have a pro-adipogenic effect or otherwise positively regulate other processes relevant to adipocyte function. To our surprise, lipid accumulation in adipocytes was increased instead of the expected decrease upon knockdown of SCAMP3. This suggests that SCAMP3 has an attenuating effect on adipogenesis and/or adipocyte function. The observed increased expression of adipocyte-enriched genes FABP4, PLIN, ADIPOQ after the treatment by siSCAMP3 further strengthen the findings of SCAMP3 as a negative regulator of adipogenesis.

Admittedly the observed effects on the lipid accumulation after SCAMP3 knockdown were not large. However, knockdown of SCAMP3 affected the expression of genes important for adipocyte phenotype by 50 to 80% at early stage of differentiation. Lipid accumulation was measured 10 days after the siRNA transfection, e.g. late in differentiation. We consider the anti-adipogenic role of SCAMP3 is relevant given into account a comparison that about 70–75% knock of an essential pro-adipogenic gene PPARG resulted in 50% decrease of the lipid accumulation.

The functional relevance of SCAMP3 in adipogenesis is also supported by its relative enrichment in adipocytes, compared to progenitors and monocytes/macrophages, according to cell-type specific transcriptome data in human adipose tissue (GEO GSE80654 and Supplemental Fig. 3). It is reasonable to speculate the role of SCAMP3 as an endogenous limiter of adipogenesis driven by PPARG, whereby SCAMP3 behaves like an anticipatory modulator that prevents, from an early stage, adipogenesis from occurring prematurely without proper stimulation, or from overshooting the optimal maturation extent. Conversely, excessive activation of SCAMP3 may hinder adipogenesis partially or even completely. The PPARG/SCAMP3 axis could be important for maintaining a balance between hypertrophic and hyperplastic expansion of adipose tissue in obesity. To evaluate the possible importance of SCAMP3 for *in vivo* situation, we performed correlation analyses between multiple subcutaneous adipose phenotypes and expression of SCAMP3 and PPARG. The data confirm the counteracting roles of PPARG and SCAMP3; the expression of both correlated in the opposite manner with a favorable adipose phenotype. This supports the hypothesis that SCAMP3 is an important factor in adipocyte function at least in women. Gender differences in adipose tissue gene expression are reported^27^ and therefore results with men might differ.

Herein we focused on two targets of the FFLs regulated by miR-27a/b-3p and PPARG. It is possible that several of the remaining eight targets also have a role in adipogenesis. Additionally, in this study we only considered PPARG as TF, and set quite strict criteria on expression and correlation for identifying candidate FFLs. By relaxing the thresholds and including other TFs it might be possible to identify numerous additional FFLs with regulatory relationships between miRNAs, TFs and downstream targets during adipogenesis. Finally, only FFLs with the miRNA upstream of both TF and target gene were considered. Other FFL types, for instance with the TF downstream of the miRNA and its target, may also have implications on adipogenesis and remain to be explored in this context.

In summary, this study demonstrates that miR-27a/b-3p regulates SCAMP3 through a FFL containing PPARG. In addition, SCAMP3 is a novel target for miR-27a/b-3p with anti-adipogenic function by itself affecting expression of adipogenic genes early in differentiation. Therefore, SCAMP3 together with previously described miR-27 represents a new class of adipogenic inhibitors, which may play a role in the development of hypertrophic adipose tissue in obesity.

## Materials and Methods

### CAGE time course data

Transcriptomic mapping was performed by cap analysis gene expression (CAGE). Libraries were prepared, sequenced, and processed as described^7,28^. Briefly, libraries were prepared using 5 μg of total RNA and sequenced on the HeliScope Single Molecule Sequencer platform. Three replicates were used per time point. CAGE reads were filtered for ribosomal RNA and mapped to the human genome (hg19) using Delve (http://fantom.gsc.riken.jp/software/). FANTOM5 CAGE data is available at the DNA Data Bank of Japan (DDBJ) under accession numbers DRA000991, DRA002711, DRA002747 and DRA002748^29^.

### miRNA time course data

Short RNA libraries were prepared and processed as described^13^. Briefly, libraries were prepared using the same RNA samples from which CAGE libraries were produced. The short RNA libraries were sequenced using the Illumina HiSeq. 2000 sequencer. Sequences were filtered for ribosomal content and mapped to the genome assembly hg19 (human) using the Burrows-Wheeler Alignment (BWA) tool^30^. The miRBase database^31^ was used as our reference set of known miRNAs. Short RNA data is available at DDBJ under accession number DRA003769.

### Adipocyte cell culture, microRNA and small interfering RNA transfection

Isolation, growth and differentiation of human adipose-derived stem cells (hASCs) was carried out as previously described^32^. Images of lipid accumulation and measurements of marker gene expression can be found in the Supplementary Material^28^.

In order to mimic the function of endogenous miRNAs, hASCs were transfected with miR-27a/b-3p miRIDIAN miRNA mimics or mimic non-targeting negative control (Dharmacon, Thermo Fisher Scientific, Lafayette, CO) at day 8 of differentiation using Neon electroporator (Invitrogen, Carlsbad, CA) and according to the manufacturer’s protocol. Briefly, for one transfection reaction 1 million of hASCs suspended in 90 µl of R buffer was mixed with 200 pmol of miRNA mimics and transfection was performed using 100 µl electroporation tip. Electroporation conditions were 1400 Volts, 20 ms width and 2 pulses. Following electroporation the cells were plated to 24-well plates at the density of 100.000 cells/well in 500 µl medium and giving the final concentration of miRNA of 40 nM. The next day medium was replaced to the fresh medium and then subsequently every second day. The cells were incubated for the following 48 h–120 h when RNA, medium and proteins were collected.

Proliferating hASCs were transfected with miRNA mimics using conditions 1150 Volts, 30 ms width, 2 pulses and plated to 24-well plates in 500 µl medium at the density of 57.000 cells/well. The cells were incubated for the following 48 h when RNA was collected.

hASCs were reverse transfected 24 h before induction of adipogenesis using ON-TARGETplus SMARTpool siRNAs targeting SCAMP3 or nontargeting siRNA control #1 (Dharmacon) as previously described^33^. The RNA and medium were collected at days 2, 6 and 9 after the induction of differentiation.

### RNA extraction, cDNA synthesis and qRT-PCR

miRNeasy kit (Qiagen, Hilden, Germany) was used to extract total RNA from adipocytes transfected with miRNA mimics. Synthesis of cDNA was performed using miScript II RT-Kit and miScript HiFlex Buffer (Qiagen) enabling detection of multiple miRNAs and mRNAs from a single cDNA preparation. In these samples qRT-PCR of coding genes or miRNAs was performed using commercial Taqman probes (Applied Biosystems, Foster City, CA) or miScript Primer Assays (Qiagen), respectively.

NucleoSpin RNA II kit (Macherey-Nagel, Düren, Germany) was used to extract total RNA from adipocytes transfected with siRNA. Reverse transcription was performed using the iScript cDNA synthesis kit (Qiagen) and random hexamer primers (Invitrogen, Carlsbad, CA). Quantitative RT-PCR of coding genes was performed using commercial TaqMan probes (Applied Biosystems).

Concentration and purity of RNA were measured using a Nanodrop ND-1000 Spectrophotometer (Thermo Fisher Scientific). Relative gene expression was calculated using the 2^−ΔCt^ method^34^. 18 s, LRP10 and SNORD68 were used as internal controls for the normalization of, respectively, coding genes and miRNAs. The expression of the reference genes did not differ between groups. Expression of *18s* was stable during adipocyte differentiation in contrast to the expression of *LRP10*.

### Analysis of protein expression

The hASCs were transfected with miRNA mimics at day 8 of differentiation as described above. 48 and 120 h postransfection the cells were collected to prepare total protein lyzates in RIPA buffer and concentrationas was measured using BCA assay (Thermo Fisher Scientific). 20 µg of total protein was separated by Mini-PROTEAN® TGX Stain-Free™ Precast Gels (Bio-Rad) allowing direct protein visualization in the gel and after the transfer on to the membrane. Protein transfer to the PVDF membrane was performed using Trans-Blot Turbo Transfer System (Bio-Rad). The membranes were blocked in 3% ECL Advance Blocking Agent (GE Healthcare, Little Chalfont, UK). Primary antibodies SCAMP3‐rabbit IgG (Proteintech, Rosemont, IL, USA) used at a concentration of 1:3000. Secondary rabbit‐IgG/goat‐IgG antibodies conjugated to horseradish peroxidase were used (Sigma‐Aldrich). Proteins were detected by chemiluminescence using the ECL Select Western Blotting Detection Kit (GE Healthcare) in the Chemidoc MP (Bio-Rad) and quantified using ImageLab software (Bio-Rad).

### Luciferase reporter assay

Empty Firefly/Renilla Duo-Luciferase reporter vector and vector containing 3′-UTR of ABCA1 or SCAMP3 were obtained from GeneCopoeia (Rockville, MD). The luciferase reporter assay in 3T3-L1 cells was performed as described previously^35^.

### Quantification of neutral lipids and cell number during adipogenesis

Lipid accumulation was quantified at differentiation day 9. hASCs differentiated *in vitro* were washed with PBS and fixed with 4% paraformaldehyde solution for 10 min at room temperature. Fixed cells were washed with PBS and stained with Bodipy 493/503 (0.2 µg/mL; Molecular Probes, Thermo Fisher Scientific) and Hoechst 33342 (2 µg/mL; Molecular Probes) for 20 min at room temperature. After washing with PBS, accumulation of intracellular lipids (Bodipy) and cell number (Hoechst) were quantified with CellInsight™ CX5 High Content Screening (HCS) Platform (Thermo Fischer Scientific). Total Bodipy fluorescence (lipid droplets) was normalized to the amount of nuclei in each well.

### Clinical cohort

We made a retrospect analysis of data from a published cohort of 56 women described in detail^35^. The clinical measures included body mass index (BMI), percentage body fat evaluated by bioimpendance, waist circumference, waist-hip ratio and homeostasis model assessment of insulin sensitivity (HOMA). Measurements of subcutaneous adipose tissue include fat cell volume, abdominal lipolysis activity (release of glycerol from adipose tissue) and insulin stimulated lipogenesis^35^. In addition we measured release of the inflammatory proteins interleukin 6 and tumor necrosis factor alpha in the adipose tissue incubated *in vitro* as described in detail^36^.

### Statistics

Values are mean ± standard deviation (SD). Standard statistical tests were used including t-test, single or multiple regression and one-way ANOVA as indicated in figure/table legends using StatView software (Abacus Concepts Inc, Berkley, CA).

## Supplementary information

Supplementary figures 1–3

## References

[1] Rosen ED, MacDougald OA (2006). Adipocyte differentiation from the inside out. Nat Rev Mol Cell Biol.

[2] Lefterova MI, Lazar MA (2009). New developments in adipogenesis. Trends Endocrinol Metab.

[3] Rosen ED, Spiegelman BM (2014). What we talk about when we talk about fat. Cell.

[4] Zuo Y, Qiang L, Farmer SR (2006). Activation of CCAAT/enhancer-binding protein (C/EBP) alpha expression by C/EBP beta during adipogenesis requires a peroxisome proliferator-activated receptor-gamma-associated repression of HDAC1 at the C/ebp alpha gene promoter. J Biol Chem.

[5] Engin AB (2017). MicroRNA and Adipogenesis. Adv Exp Med Biol.

[6] Lai X, Wolkenhauer O, Vera J (2016). Understanding microRNA-mediated gene regulatory networks through mathematical modelling. Nucleic Acids Res.

[7] Ehrlund A (2017). Transcriptional Dynamics During Human Adipogenesis and Its Link to Adipose Morphology and Distribution. Diabetes.

[8] Alon U (2007). Network motifs: theory and experimental approaches. Nat Rev Genet.

[9] Tsang J, Zhu J, van Oudenaarden A (2007). MicroRNA-mediated feedback and feedforward loops are recurrent network motifs in mammals. Mol Cell.

[10] Cheng C (2011). Construction and analysis of an integrated regulatory network derived from high-throughput sequencing data. PLoS Comput Biol.

[11] Re A, Cora D, Taverna D, Caselle M (2009). Genome-wide survey of microRNA-transcription factor feed-forward regulatory circuits in human. Mol Biosyst.

[12] Shalgi R, Brosh R, Oren M, Pilpel Y (2009). & Rotter, V. Coupling transcriptional and post-transcriptional miRNA regulation in the control of cell fate. Aging.

[13] de Rie D (2017). An integrated expression atlas of miRNAs and their promoters in human and mouse. Nat Biotechnol.

[14] Garcia DM (2011). Weak seed-pairing stability and high target-site abundance decrease the proficiency of lsy-6 and other microRNAs. Nat Struct Mol Biol.

[15] Oki, S. *et al*. ChIP-Atlas: a data-mining suite powered by full integration of public ChIP-seq data. *EMBO Rep* **19**, 10.15252/embr.201846255 (2018). 10.15252/embr.201846255 PMC6280645 30413482

[16] Portius D, Sobolewski C, Foti M (2017). MicroRNAs-Dependent Regulation of PPARs in Metabolic Diseases and Cancers. PPAR Res.

[17] Le Lay S (2003). Regulation of ABCA1 expression and cholesterol efflux during adipose differentiation of 3T3-L1 cells. J Lipid Res.

[18] Cuffe H (2018). Targeted Deletion of Adipocyte Abca1 (ATP-Binding Cassette Transporter A1) Impairs Diet-Induced Obesity. Arterioscler Thromb Vasc Biol.

[19] Aoh QL, Castle AM, Hubbard CH, Katsumata O, Castle JD (2009). SCAMP3 negatively regulates epidermal growth factor receptor degradation and promotes receptor recycling. Mol Biol Cell.

[20] Falguieres T, Castle D, Gruenberg J (2012). Regulation of the MVB pathway by SCAMP3. Traffic.

[21] Riba A, Bosia C, El Baroudi M, Ollino L, Caselle M (2014). A combination of transcriptional and microRNA regulation improves the stability of the relative concentrations of target genes. PLoS computational biology.

[22] Lin Q, Gao Z, Alarcon RM, Ye J, Yun Z (2009). A role of miR-27 in the regulation of adipogenesis. FEBS J.

[23] Karbiener M (2009). microRNA miR-27b impairs human adipocyte differentiation and targets PPARgamma. Biochem Biophys Res Commun.

[24] Kang T (2013). MicroRNA-27 (miR-27) targets prohibitin and impairs adipocyte differentiation and mitochondrial function in human adipose-derived stem cells. J Biol Chem.

[25] Zhu Y (2014). miR-27 inhibits adipocyte differentiation via suppressing CREB expression. Acta Biochim Biophys Sin (Shanghai).

[26] Chen SZ (2015). miR-27 impairs the adipogenic lineage commitment via targeting lysyl oxidase. Obesity (Silver Spring).

[27] Karastergiou K (2013). Distinct developmental signatures of human abdominal and gluteal subcutaneous adipose tissue depots. J Clin Endocrinol Metab.

[28] Arner E (2015). Transcribed enhancers lead waves of coordinated transcription in transitioning mammalian cells. Science.

[29] Lizio M (2019). Update of the FANTOM web resource: expansion to provide additional transcriptome atlases. Nucleic acids research.

[30] Li H, Durbin R (2009). Fast and accurate short read alignment with Burrows-Wheeler transform. Bioinformatics.

[31] Kozomara A, Griffiths-Jones S (2014). miRBase: annotating high confidence microRNAs using deep sequencing data. Nucleic acids research.

[32] Pettersson AM (2013). LXR is a negative regulator of glucose uptake in human adipocytes. Diabetologia.

[33] Jiao H (2016). Whole-Exome Sequencing Suggests LAMB3 as a Susceptibility Gene for Morbid Obesity. Diabetes.

[34] Livak KJ, Schmittgen TD (2001). Analysis of relative gene expression data using real-time quantitative PCR and the 2(-Delta Delta C(T)) Method. Methods.

[35] Arner E (2012). Adipose tissue microRNAs as regulators of CCL2 production in human obesity. Diabetes.

[36] Arvidsson E (2004). Effects of different hypocaloric diets on protein secretion from adipose tissue of obese women. Diabetes.

