# Supplementary material for: MicroRNA-27a/b-3p and PPARG regulate SCAMP3 through a feed-forward loop during adipogenesis
Source: Sci Rep. 2019 Sep 25;9:13891. doi: 10.1038/s41598-019-50210-3 (PMC6761119; doi:10.1038/s41598-019-50210-3)

Title: MicroRNA-27a/b-3p and PPARG regulate SCAMP3 through a feed forward loop during adipogenesis

Authors: Agné Kulyté, Kelvin Ho Man Kwok, Michiel de Hoon, Piero Carninci, Yoshihide Hayashizaki, Peter Arner, Erik Arner

Supplemental figure 1. miR-27a-3p and miR-27b-3p regulate SCAMP3 in proliferating hASCs.

miR-27a-3p and miR-27b-3p were overexpressed in proliferating hASCs and expression of SCAMP3 was assessed by RT-qPCR. Expression of genes was normalized to the reference gene LRP10. Results are based on two biological/independent experiments. Results were analyzed using t-test and presented in fold change ± SD relative to negative control of a corresponding time point (Neg C). ***P < 0.005, **P < 0.01, *P < 0.05.

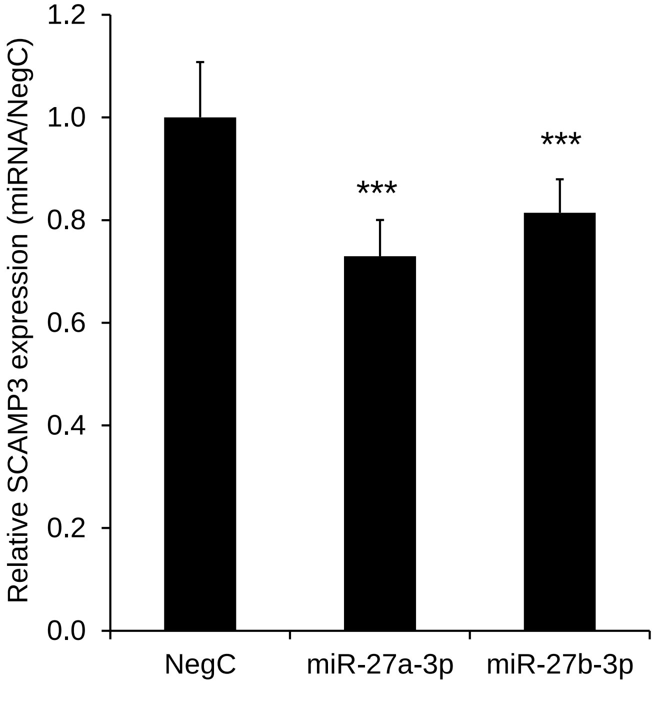

Supplemental figure 2. Images of hASCs at day 9 transfected with siRNA NegC, siSCAMP3 and siPPARG and differentiated *in vitro*.

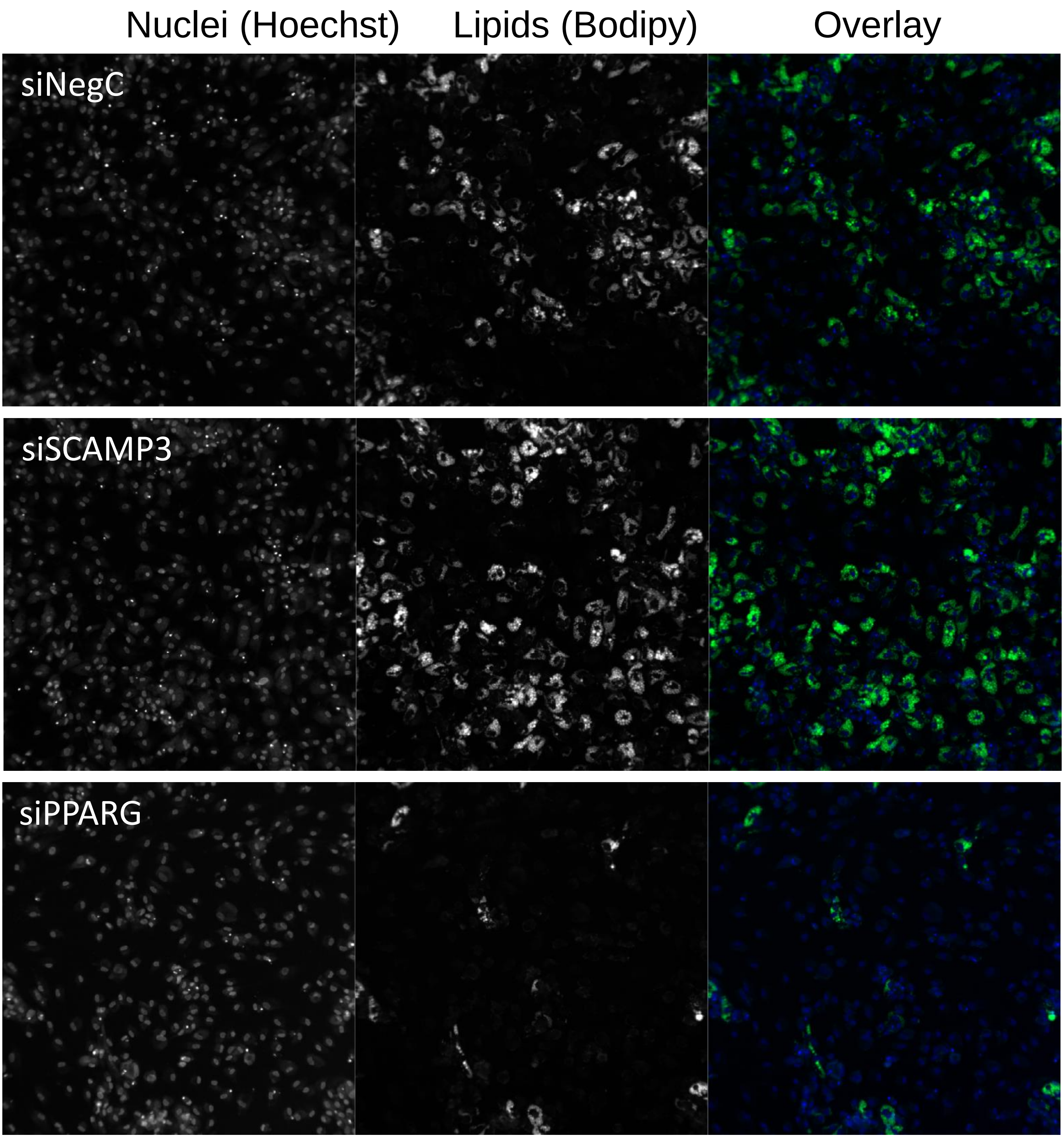

Supplemental figure 3. Expression of SCAMP3 in FASC sorted human adipose tissue stromal vascular fraction. Data retrieved from URL https://www.ncbi.nlm.nih.gov/geo/query/acc.cgi?acc=GSE80654.

Adipose tissue from 6 patients was collagenase treated and adipocytes separated from the stromal vascular fraction (SVF). SVF was then FACS sorted for the following fractions CD45-/CD34+/CD31+ (endothelial), CD45-/CD34+/CD31- (progenitor), CD45+/CD14+ (monocyte/macrophage), CD45+/CD14-(Leukocyte). RNA was isolated from adipocytes, SVF, progenitor, macrophage/monocyte and leukocyte fractions and analyzed on the Affymetrix Human Transcriptome 2.0 array. ***P < 0.005.

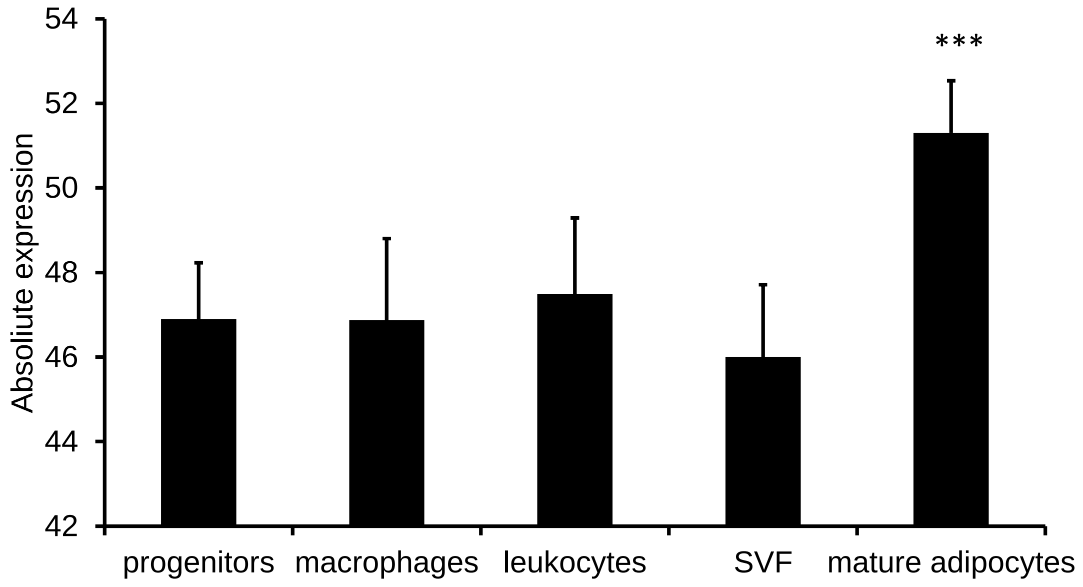

Supplement: Supplementary file 1 — Supplementary figures 1–3 [file 41598_2019_50210_MOESM1_ESM.pdf]
